# Influence of lateral cephalometric radiographs on extraction decision in skeletal class I patients

**DOI:** 10.1186/1746-160X-9-36

**Published:** 2013-12-04

**Authors:** Banu Dinçer, Enver Yetkiner, Isil Aras, Thomas Attin, Rengin Attin

**Affiliations:** 1 Department of Orthodontics, Faculty of Dentistry, University of Ege, Izmir, Turkey; 2 Center of Dental Medicine, Clinic for Preventive Dentistry, Periodontology and Cariology, University of Zürich, Zürich, Switzerland; 3 Center of Dental Medicine, Department of Orthodontics and Pediatric Dentistry, University of Zürich, Zürich, Switzerland

## Abstract

**Background:**

Radiographic examination is considered ‘justified’ only when detection of a condition that would change the mechanisms and timing of treatment is possible. Radiographic safety guidelines have restricted the indication of lateral cephalometric radiographs (LCRs) to presence of distinct skeletal Class II or Class III. However, they are taken routinely in clinical practice and considered to be part of the ‘gold’ standard for orthodontic diagnosis. Therefore, the aim of this study was to test the null hypothesis that lateral cephalometric radiograph (LCR) evaluation would not alter the extraction/non-extraction decision in orthodontic treatment planning of skeletal Class I patients.

**Materials and methods:**

Intraoral and extraoral photographs, dental casts and extraoral radiographs of 60 skeletal Class I patients were prepared digitally for assessment using a presentation software. One experienced (EO) and inexperienced orthodontist (IO) was asked to decide on extraction or non-extraction on a Likert-type linear scale for treatment planning. This procedure was repeated 4 weeks later with a mixed order of patients and the LCRs being omitted. Kappa, Weighted Kappa (WK) and McNemar scores were computed to test decision consistency and Bland-Altman plots together with 95% limits of agreement were used to determine measurement accuracy and presence of systematic bias.

**Results:**

Both EO (WK = 0.67) and IO (WK = 0.64) had good level of decision agreement with and without LCR evaluation. EO did not present a shift towards extraction nor non-extraction with LCR evaluation (McNemar = 0.999) whereas IO showed a tendency to extraction (McNemar = 0.07) with LCR data. Including LCR evaluation created a systematic inconsistency between EO and IO (Line of equality = 0.8, Confidence interval = 0.307-0.707).

**Conclusions:**

Lateral cephalometric radiograph evaluation did not influence the extraction decision in treatment planning of skeletal Class I patients. Reconsidering the necessity of lateral cephalograms in orthodontic treatment of skeletal Class I patients may reduce the amount of ionizing radiation. Key words: Lateral cephalometric radiograph, extraction, treatment planning, skeletal Class I.

## Introduction

Orthodontic diagnosis and treatment planning is based on comprehensive information obtained from the patient. These data usually consist of detailed medical history, clinical examination, study models, extraoral radiographs (panoramic-lateral cephalometric), intraoral radiographs (bitewing-periapical) and photographs (intraoral/extraoral) [1,2]. Among these tools, diagnostic radiation is the only critical application that might be harmful for the patient due to its stochastic effects such as increasing the risk of fatal/non-fatal cancer and hereditary changes [3,4].

Radiographic examination is considered ‘justified’ only when detection of a condition that would change the mechanisms and timing of treatment is possible [5]. It is contra indicatory to prescribe diagnostic radiographs when clinical signs and symptoms are not present [6]. In addition to these prerequisites, it is essential that the methods used in assessing the obtained images have high inter- and intra-examiner reliability as well as valid estimation levels for the malformation it is intended to identify [5]. Still, lateral cephalometric radiographs (LCRs) are considered to be part of the ‘gold’ standard for diagnosis at the start of orthodontic treatment [1,2,6] and taken routinely in clinical practice [7,8]. It has been reported that roughly three LCRs, depending on treatment duration, patient gender, age and presence of surgical component, are prescribed during average orthodontic treatment [8].

Primary aims of LCR use are summarized as follows: assessment of pathologies and/or deviations from normal cranio-facial anatomy; growth estimation in terms of direction and magnitude; assessment of treatments and their effects on normal growth; comparisons of different treatment outcomes at different developmental stages and with different facial types [1,2,6]. In accordance, the diagnostic indications that were accepted by the European Guidelines on Radiation Protection in Dental Radiology for requesting LCRs were defined as the presence of distinct skeletal Class II or Class III pattern and Grade 4 of the Index of Orthodontic Treatment Need Dental Health Component (IOTN DHC) [9]. In addition to these pre-treatment diagnostic conditions, determination of lower anterior teeth proclination following functional treatment, presurgical planning for orthognathic cases and determination of lower incisor positions that would change the finishing mechanics or retention regime were the LCR indications that could be requested in order to assess the treatment effects [9].

Despite the presence of these guidelines and regulations, LCRs are continued to be frequently requested due to the reason that cephalometric analysis is the only practical quantitative method that permits the investigation and evaluation of the spatial relationships between cranial and dental structures. This also allows its use for diagnosis and treatment planning as well as for a tool of research [10-12]. However, LCRs were found to have a major impact on diagnosis but a minor impact on treatment planning. Only 7-24% of treatment plans were reported to change following radiographic evaluation [12,13]. LCRs were shown to have no influence on treatment planning prior to late mixed dentition even in the presence of skeletal discrepancies [14]. Parallel to these results, dental casts and initial clinical examinations alone were reported to provide adequate information for orthodontic treatment planning [11-16].

Another reason for requesting LCRs on a routine basis is to justify teeth extraction, as being one of the most critical diagnostic decisions in clinical orthodontics [17,18]. Although the diagnostic data and treatment plan are unique for each patient, orthodontists seem to have tendencies towards extraction or non-extraction decisions [18]. Extraction rates differ greatly among orthodontists and data obtained from LCRs are usually used to support the extraction/non-extraction decision, which is mainly influenced by types of malocclusions, possible treatment techniques and expected treatment outcomes [16-18].

These conflicting results generate the question whether it is really influential to take LCRs with respect to the indications presented on the guidelines. Therefore, the aim of this study was to investigate whether LCRs alter orthodontic treatment planning in terms of extraction decision in skeletal Class I patients. The null hypothesis tested was that lateral cephalometric radiograph evaluation of skeletal Class I patients would not alter the extraction/non-extraction decision in orthodontic treatment planning.

## Materials and methods

### Study design

Two diagnostic record conditions, (1) intraoral and extraoral photographs, digital dental models, panoramic x-rays, LCRs and (2) identical content of records excluding LCRs and relevant data were involved in this study. First sets of records were anonymously presented to one experienced (EO, 20 years of experience) and one inexperienced (IO, 4 years of experience) orthodontist for extraction/non-extraction assessment. Each orthodontist noted his or her evaluation on a linear Likert-type scale individually [18]. This procedure was repeated 2 weeks later with the same set of records for internal reliability calculation. Four weeks later, using the second set of records in a mixed order excluding the LCRs, the procedure was repeated. Consistencies of the decisions were evaluated statistically.

### Subjects

Patient files, which were considered for treatment at University of Ege, Faculty of Dentistry, Department of Orthodontics between the years 2010 and 2012 were evaluated for inclusion in this cohort study. Two experienced clinical instructors (B.D. and R.A) screened 500 patient records and 60 files (20 minor crowding, 20 moderate crowding, 20 major crowding) meeting the inclusion criteria were elected. Preliminary eligibility criteria for inclusion in the sample were [19]:

• Caucasian females and males between 12–18 years of age

• Presence of permanent dentition

• Absence of craniofacial and dento-alveolar malformations

• 1 < ANB° < 5

• 22 < FMA° < 28

• 70 < Z° < 80

Case records with space discrepancy less than 4 mm, 4 to 7 mm and more than 7 mm were defined as minor, moderate and major crowding cases, respectively [1,2,19].

### Data presentation

Patient files were numbered and the identification data were concealed. All data including digital dental models (space analysis and Bolton ratio), extra- and intraoral photographs, extra- and intraoral radiographs, cephalometric tracings (Downs, Steiner, Tweed analyses) and chart information were presented digitally and were independently scored by the two orthodontists, who use LCRs routinely for treatment planning. The orthodontists differed only in years of clinical experience (EO, 20 years; IO, 4 years) and did not have routine clinical preferences of extraction/non-extraction treatment planning or bracket prescriptions. Both orthodontists were unaware that they would repeat the scoring procedure with the absence of LCR on a future date. Assessment time for each patient was unlimited. Main treatment aim was defined as achieving healthy functional occlusion with facial soft tissue harmony and esthetics. Treatment methods, materials and financial conditions were not restricted. No retention definition was made. Extraction was defined as removal of minimum one permanent tooth with the exclusion of third molars. For each patient record, the orthodontists were requested to mark their decisions on a linear Likert-type scale as shown below [20]:

1. Definitely non-extraction

2. Non-extraction

3. Borderline, may or may not extract

4. Extraction

5. Definitely extraction.

### Statistical analysis

Assuming a 0.89 proportion of successes, an intra-class kappa of 0.3 and a sample size of 56 patient files were calculated to have 90% power to detect an alternative kappa of 0.9 with a 0.05 level two-sided test. Therefore, 60 cases were evaluated in order to provide more than 90% power.

Obtained categorical data were coded in Excel, analyzed with SPSS Version 20.0.02 and MedCalc Version 12.4.0. Kappa and weighted Kappa scores were computed and their relevance was assessed as follows [19]: 0–0.2: poor agreement; 0.2-0.4 fair agreement; 0.4-0.6 moderate agreement; 0.6-0.8 good agreement; 0.8-1 excellent agreement. Bland-Altman plots together with 95% limits of agreement were computed [21]. Dichotomized version of the grading scale was computed as 1–2 coded as 1 and 4–5 coded as 0. For these binary versions, Kappa and weighted Kappa as well as McNemar test were computed. Results were considered significant where p < 0.05 and as tendency where 0.05 < p < 0.1 [21,22].

## Results

Depending on the internal reliability evaluation data, computed Weighted-Kappa scores of EO and IO were 0.921 and 0.710, respectively. This indicated excellent internal reliability for EO and good intra-reliability for IO.

Equivalence level of extraction decision with and without LCR data was good and moderate for EO and IO, respectively. EO and IO presented moderate agreement between themselves for their decision with or without LCR data.

According to the dichotomous data analyzed by McNemar test, IO decided on extraction significantly more frequent than EO with LCR data (p = 0.001) and showed a tendency to extract after LCR assessment (p = 0.07).

Bland-Altman plots revealed the presence of significantly different measurement accuracy between EO and IO for assessments with LCR data Table 1.

**Table 1:** **Line of equality**, **limits of agreement**, **95**% **confidence interval** (**CI**) **and presence of systematic bias in assessments between two orthodontists**

	**Line of equality**	**Limits of agreement**	**95% CI**	**Presence of systematic bias**
EO vs IO without LCR	0.4	-3.7 / 2.9	0.175-0.655	Negative
EO vs IO with LCR	0.8	-3.8 / 2.2	0.307-0.707	Positive

Detailed Kappa, Binary Kappa, Weighted Kappa and McNemar scores as well as levels of agreements and 95% confidence intervals are shown in Tables 2 and 3. Line of equality, limits of agreement and 95% confidence intervals for Bland-Altman plots are presented in Table 4.

**Table 2:** **Mean**, **standard deviation** (**SD**), **minimum** (**Min**) **and maximum** (**Max**) **values of cephalometric measurements**

	**U1-L1**	**IMPA**	**FMA**	**U1-FH**	**FMIA**	**SNA**	**SNB**	**ANB**	**U1-SN**	**HOLDAWAY**	**L1-NB**	**S-Go/N-Me**	**WITS**	**U1-NA**	**U1-NA (mm)**	**POG-NB (mm)**	**SN-GoGN**	**JARABAK**	**L-E PLAN**	**U-E PLAN**	**Z Angle**
Mean	122.3	93.8	25.0	115.9	61.6	81.3	78.1	3.1	108	1.0	4.8	66.6	0.8	26.7	4.6	1.7	31.8	394.2	-1.9	-3.4	74.9
SD	6.8	6.0	4.2	6.2	4.3	3.1	12.2	0.9	4.7	2.5	2.0	5.0	1.9	6.1	2.5	1.9	6.1	6.1	2.5	2.7	3.1
Min	105.5	82.6	22.0	103.8	53.4	71.3	68.3	1.1	99.7	-0.3	0.2	57.2	-2.7	16.8	1.2	-2.1	20.6	382.8	-5.9	-8.8	70.0
Max	139.4	118.5	27.2	128.5	72.4	86.5	84.3	4.9	116.7	15	8.9	75.1	4	39.7	10.0	7.4	46.7	408.7	3.3	1.5	79.8

All values are angle degrees unless indicated otherwise.

**Table 3:** **Kappa**, **level of agreement**, **standard error** (**Std. error**) **and 95**% **confidence interval** (**CI**) **values of EO and IO according to their extraction decisions with and without cephalogram**

	**Kappa**	**Level of agreement**	**Std. error**	**CI 95%**
EO vs. IO without cephalogram	0.283	Fair	0.078	0.130-0.435
EO vs. IO with cephalogram	0.309	Fair	0.073	0.165-0.453
EO with and without cephalogram	0.529	Moderate	0.093	0.346-0.712
IO with and without cephalogram	0.444	Moderate	0.082	0.283-0.605

**Table 4:** **Binary Kappa**, **Weighted Kappa**, **level of agreement**, **Mc Nemar**, **standard error** (**Std. error**) **and 95**% **confidence interval** (**CI**) **values of EO and IO according to their extraction decisions with and without cephalogram**

	**Binary kappa**	**Weighted kappa**	**Level of agreement**	**Mc Nemar**	**Std. error**	**CI 95%**
EO vs. IO without cephalogram	0.415	0.404	Moderate	0.210	0.120	0.175-0.655
EO vs. IO with cephalogram	0.507	0.447	Moderate	0.001	0.100	0.307-0.707
EO with and without cephalogram	0.762	0.678	Good	0.999	0.092	0.578-0.946
IO with and without cephalogram	0.730	0.640	Good	0.070	0.087	0.556-0.904

## Discussion

In this cohort study, influence of lateral cephalograms on extraction/non-extraction decision of skeletal Class I patients was evaluated. Both the experienced and inexperienced orthodontist presented consistency in their extraction decisions with or without LCRs. Thus, the tested null hypothesis that the cephalometric evaluation would not alter the extraction/non-extraction decision in orthodontic treatment planning of skeletal Class I patients cannot be rejected.

Case selection criteria used in this study was skeletal Class I patients having three different levels of crowding (less then 4 mm, between 4 to 7 mm and more than 7 mm) with normal vertical growth pattern. Patient files complying with these conditions were selected using pre-treatment diagnostic records. The primary aim behind these settings was to include patient data that would not require the ordering of LCRs, in accordance with the European Guidelines on Radiation Protection in Dental Radiology [9]. In order to fulfill these requirements, none of the selected files presented a distinct skeletal Class II or Class III pattern nor had IOTN DHC grade of 4 or more, which also represents the majority of malocclusions [1,2,23]. The secondary aim was to narrow down the primary assessment outcome only to extraction/non-extraction decision in a group of patients with minor, moderate and major crowding, which remains the main rationale for the extraction decision, reported by orthodontists [18].

The internal reliability of the orthodontists taking part in the study was important in order to prevent varying judgments at different time-points. Both assessors (EO and IO) presented excellent and good internal reliability, respectively. This indicated relatively stable judgments at different time points using the same sets of records, which increases the probability that the findings are consistent in general. On the other hand, the low number of assessors potentially increased the risk of subjective bias for this study despite the fact that they were unaware of re-assessment procedure excluding LCRs. Nevertheless, the main aim was to compare the influence of LCR data on extraction/non-extraction decision on treatment planning, not factors related to the orthodontists’ individual interpretation variations. Therefore, the power calculation was based on the number of files to be scored rather than the number of orthodontists grading them.

When the consistency of extraction decisions with and without having the LCR data is evaluated, LCR data did not influence the extraction decision of both orthodontists and they presented good equivalence of extraction decisions with both sets of records. These findings are in accordance with previous studies evaluating the contribution of pre-treatment diagnostic radiographs to treatment planning [11,12,16,24,25]. Although not significant, the presence of LCR data seemed to influence the IO and created a tendency to shift to extraction decision according to dichotomized results. This was further supported by Bland-Altman plots, which revealed the significantly different measurement accuracy between EO and IO and the systematic bias of IO for assessments with LCR data. It could be claimed that this inconsistency arises from the different experience levels. Yet, influence of experience was not the primary aim of this study and the statistical power to assess this factor is very low with this data. Repeating a similar study with a larger number of orthodontists having different levels of experience and comparing their assessments might answer the question of whether the extraction decision is influenced by the experience levels with/without the presence of LCR data.

Extraction decision, a major irreversible factor in orthodontic treatment planning, is usually justified by information obtained from the LCRs [1,2,17,18]. The possible consequences of extraction/non-extraction treatment modalities are mainly reflected on soft tissues, vertical cranio-facial dimensions, transversal arch width, smile esthetics and probability of relapse [17,18,24,26]. Yet, results derived from studies evaluating these associations remain controversial while management of the extraction space and clinician skills are reported to be more influential. While crowding being the primary rationale for extraction, clinicians were observed to focus more on appearance-related factors that were available on study casts and facial photographs [18].

Previously, it has been reported that orthodontic diagnosis is affected majorly by the presence of LCRs whereas treatment planning is not, due to the convenience of precise skeletal diagnostic data obtained from the LCRs, which is not playing a significant role on the treatment plan. However, due to the inherent problems of traditional LCRs and inconsistent clinical information derived from these data, the validity and reproducibility of LCR assessment methods have also been called into question [1,27]. The most important problem adversely affecting the data obtained from LCRs was reported as the representation of a three-dimensional anatomic complex on a two-dimensional conventional LCR, which leads to the vertical and horizontal displacement of structures depending on their distance to the film [1,27,28]. Furthermore, inaccuracies related to radiographic projection, such as magnification-distortion problems and patient positioning errors may be present [1,27]. One can still argue the precision of distinguishing skeletal problems only by clinical examination without prescribing a LCR. However, it has been reported that orthodontists, when compared with Class II or Class III profiles could easily recognize a Class I profile. Therefore, estimation of the presence of distinct skeletal Class II or Class III in order to prescribe a LCR is likely by an orthodontist only by clinical examination [29].

Digital presentation of the cases can be considered as a factor affecting the reliability of the results adversely, since it is a great deviation from the routine diagnosis and treatment planning procedure. In particular, examining a patient from indefinite points of views under clinical circumstances and getting the three-dimensional image, certainly, will let the clinician better interpret the data that is provided. Still, these factors should not cause a major change in the decisions since patient records are the only materials that can be presented in professional platforms of diagnosis and treatment planning discussions.

One other possible drawback of the study might be that third molars were ignored in the definition of extraction. In principle, third molar extractions are involved in treatment planning regarding the posterior borders of permanent dentition. However, avoiding possible positive extraction decisions regarding only the third molars was aimed by excluding them.

## Conclusions

• Comprehensive clinical examination is important to confirm the necessity of LCRs for the patient’s specific orthodontic problem.

• Reconsidering the necessity of lateral cephalograms depending on their diagnostic validity and benefit in orthodontic treatment of skeletal Class I patients may reduce the amount of ionizing radiation.

### Ethical approval

The study was approved by the University of Ege Research Ethics Committee (Reference number B.30.2.EGE.0.20.05.00/EY/13-9/10.

## Abbreviations

LCR: Lateral cephalometric radiograph; IOTN DHC: Index of orthodontic treatment need dental health component; EO: Experienced orthodontist; IO: Inexperienced orthodontist.

## Competing interests

The authors declare that they have no competing interests.

## Authors’ contributions

BD carried out the subject selection procedure and participated in the preparation of data for presentation to EO and IO. EY participated in the design of the study, performed the statistical analysis and drafted the manuscript. IA helped the coordination of scoring procedures and proofread the manuscript. TA helped in the study design and proofread the manuscript. RA designed the study, carried out the subject selection procedure and proofread the manuscript. All authors read and approved the final manuscript.
